# Transarterial Chemoembolization (TACE) Combined with Sorafenib in Treatment of HBV Background Hepatocellular Carcinoma with Portal Vein Tumor Thrombus: A Propensity Score Matching Study

**DOI:** 10.1155/2019/2141859

**Published:** 2019-07-28

**Authors:** Jia Yuan, Xin Yin, Bei Tang, Hui Ma, Lan Zhang, Lixin Li, Rongxin Chen, Xiaoying Xie, Zhenggang Ren

**Affiliations:** ^1^Liver Cancer Institute, Department of Hepatic Oncology, Zhongshan Hospital, Fudan University, 180 Fenglin Road, Shanghai 200032, China; ^2^Key Laboratory of Carcinogenesis and Cancer Invasion, Ministry of Education, 180 Fenglin Road, Shanghai 200032, China

## Abstract

**Objectives:**

Hepatocellular carcinoma (HCC) with portal vein tumor thrombus (PVTT) remains a challenge in management. Transarterial chemoembolization (TACE) has been used for patients with PVTT but efficiency was limited with a median overall survival of 4 to 6.1 months. The aim of this study is to evaluate the efficiency of TACE combined with sorafenib in HBV background HCC with PVTT.

**Methods:**

A total of 498 patients were enrolled in the study including 69 patients who received TACE combined with sorafenib and 429 patients treated with TACE alone between January 1st, 2008, and April 30st, 2014. Using the 1:2 propensity score matching, 138 well-balanced patients were enrolled. Overall survival (OS) was compared between the two groups. The Kaplan-Meier method was used to evaluate the OS, and the differences between groups were analyzed with a log-rank test.

**Results:**

TACE combined with sorafenib improved the OS of the patients compared with TACE alone (13.0 vs 6.0 months, p<0.001). After propensity score matching, the median OS of combination therapy and TACE were 13.0 and 7.0 months, respectively (p=0.001). Subgroup analysis revealed that the patients younger than 60 years old, male patients, AFP more than 400ng/ml, tumor size more than 5cm, or type III/IV PVTT had OS benefits from TACE combined with sorafenib.

**Conclusions:**

Compared with TACE therapy alone, TACE combined with sorafenib could improve OS in HBV background HCC patients with PVTT. The patients who are younger, male, or with more tumor burden may benefit more from combination therapy.

## 1. Introduction

Hepatocellular carcinoma (HCC) is the sixth most prevalent cancer and the second most frequent cause of cancer mortality worldwide with nearly 780,000 new cases annually [1]. Most patients were found at advanced stage with portal vein tumor thrombus (PVTT) or distant metastasis with about 44%-62.2% of incidence combined with PVTT when diagnosed [2]. Plenty of studies have shown that PVTT was a very important independent risk factor in the prognosis of HCC, which can lead to not only tumor progression, but also portal hypertension and further triggered hepatic failure; therefor, PVTT is a challenge for clinical management in HCC [3].

Currently, the best treatment of HCC with PVTT remains controversial. According to Barcelona Clinic Liver Cancer (BCLC) stage, HCC with PVTT was grouped as stage C, and targeted drug sorafenib was the standard treatment method. Sorafenib is a multikinase inhibitor, which is used to treat HCC through the mechanisms of antiproliferation via blocking MAPK signaling and antiangiogenesis via inhibiting VEGFR and PDGFR [4, 5]. However, in Asia, transarterial chemoembolization (TACE) was also used to treat HCC patients with PVTT, which has been demonstrated to be effective and safe in some studies [6, 7]. In this study, propensity score matching analysis was used to reveal the benefits of TACE combined with sorafenib in HCC patients with PVTT.

## 2. Materials and Methods

### 2.1. Patients

**Figure 1:**
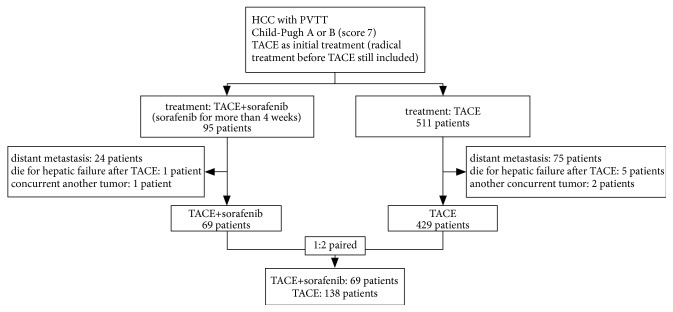
Flowchart for patients' enrollment.

This study protocol was approved by the Ethics Committee of our hospital and in accordance with the Helsinki Declaration for the ethical principle for medical research involving human subjects. There were 606 HCC patients with portal vein tumor thrombus treated in the Department of Hepatic Oncology of our hospital from January 1, 2008, to April 30, 2014. The patients were enrolled based on the following criteria: (1) diagnosis of HCC with pathology or confine to noninvasive diagnosis based on AASLD HCC diagnosis criteria; (2) imageological diagnosis with portal vein tumor thrombus; (3) with liver function of Child-Pugh A or B (score 7); (4) TACE as initial treatment at the diagnosis, radical treatment such as surgical resection and radiofrequency ablation (RFA) before TACE still included; (5) concurrent combined with sorafenib treatment more than 4 weeks. The patients with distant metastasis who died within 30 days after TACE due to hepatic failure or other major complications, or another concurrent malignant tumor, were excluded from the study. Finally, there were 498 patients enrolled including 69 patients who underwent TACE combined with sorafenib and 429 patients received TACE alone. The flowchart was shown in Figure 1.

### 2.2. TACE and Combination with Sorafenib

TACE procedure was conducted according to the standard procedure of our department. In brief, 5-Fr RH catheter was used to catheterize the celiac artery and then angiography was performed to identify the tumor feeding artery. Following perfusion of chemotherapy drugs such as 5-fluorouracil 1.0 g and oxaplatin 150 mg, super selective catheterizations were conducted to localize the catheters as close to the tumor as possible. Chemoembolization was conducted with 5-20 mL of lipiodol mixture containing 10mg Mitomycin C or 30mg Epirubicin infused slowly via the catheter.

Sorafenib was administered a few days later following TACE as liver function and blood routing were permitted. The initial sorafenib oral dose was 400 mg twice daily. The dosage of sorafenib could be reduced with grade of 200mg as the patients experienced grade II adverse events (AE) according to “NCI Common Terminology Criteria Adverse Events (CTCAE) 3.0.” Sorafenib therapy would be stopped as the grade III AE was encountered till the AE completely recovered and administration was restored at a lower dosage than before.

### 2.3. Follow-Up

All patients were evaluated one month after TACE and the repeated TACE would be performed as demand with an interval of one month and half or two months. As complete response was reached, the patients were followed up at an interval of two or three months with liver function, AFP, and enhanced CT or MRI. Patients who were administered sorafenib were followed up by conducting assays of liver and renal function, blood routing, and coagulation function each month. Sorafenib would be continued for the patients until the patients suffered from intolerant AE or no benefit was obtained as judged by the physician in the case of tumor progression. All patients were followed up until April 30, 2017.

### 2.4. Propensity Score Matching

A propensity score was constructed to account for potential bias between the groups who underwent TACE combined with sorafenib and TACE alone. All covariates related to the prognosis (P<0.10) were included in a logistic regression model. These variables included age, sex, AFP, Child-Pugh classification, tumor number, tumor size, and grade of portal vein invasion. One patient who underwent TACE combined with sorafenib was matched to two patients who underwent TACE alone. To assess the adequacy of the constructed propensity score, covariate balance was tested within quintiles of the propensity score and was found to be well balanced.

### 2.5. Statistical Analyses

Continuous variables were showed as mean ± standard deviation and compared using T test, approximate t test, or Mann-Whitney rank sum test according to the normality and homogeneity. The endpoint of the study was overall survival (OS), defined as the time from the dates of receiving first TACE therapy to the death or date of census. Categorical variables were showed as frequency and compared using chi-square test. Kaplan–Meier method was used to evaluate the OS and the difference between groups was analyzed with log-rank test. P values < 0.05 were considered to be statistically significant.

## 3. Results

### 3.1. The Characteristics of the Patients in Groups Who Underwent TACE Combined with Sorafenib and TACE Alone

The characteristics of patients who underwent TACE combined with sorafenib and TACE alone were showed in Table 1. The median age was 51 (range from 21 to 79 years old) and 51 (range from 18 to 84 years old) in combined sorafenib group and TACE alone group, respectively. In combined with sorafenib group, the patients had lower level of AFP (p=0.034), lower level of tumor size (p=0.043), and less type I/II PVTT (p=0.002). The median treatment duration of sorafenib was 7.0 months (1.0-40.5 months).

### 3.2. Overall Survival

**Figure 2:**
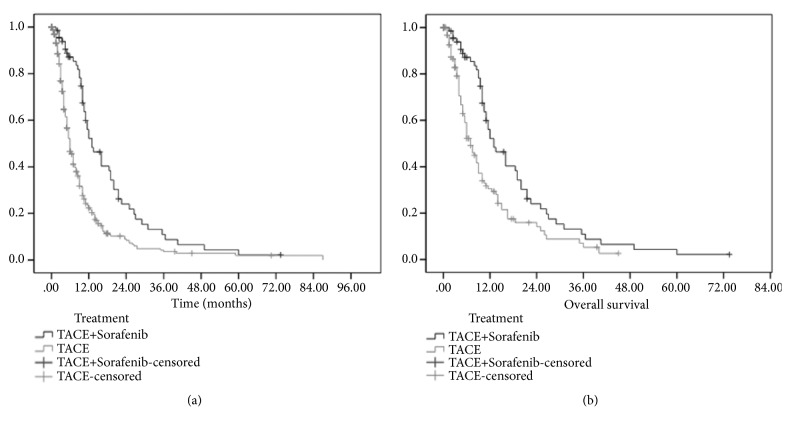
(a) Kaplan-Meier survival curves for comparison of OS between TACE + sorafenib group and TACE group (P<0.001). (b) Kaplan-Meier survival curves for comparison of OS between TACE + sorafenib group and TACE group after propensity score matching (P=0.001).

All patients were followed up until April 30, 2017, with a median follow-up duration of 5 months (range, 1 to 87 months). For the patients who underwent TACE combined with sorafenib, the median TACE procedure was 3 times (range, 1 to 11 times), and for the patients who underwent TACE alone, the median TACE was times 2 (range, 1 to 11 times). The median OS of TACE combined with sorafenib group was 13.0 months (95%CI, 9.2-16.8 months), compared with 6.0 months (95%CI, 5.4-6.6 months) for patients of TACE alone group (p<0.001) (Figure 2(a)).

As the 1 to 2 matching, there were 69 patients of TACE combined with sorafenib and 138 patients of TACE alone underwent matching analysis. As showed in Table 2, after propensity score matching, there was no significant difference between the two groups in the baseline characteristics. The median OS were 13.0 months (95%CI, 9.2-16.8 months) and 7.0 months (95%CI, 5.3-8.7 months) in patients with TACE combined with sorafenib and that with TACE alone, respectively, and there were significant differences between groups (p=0.001) (Figure 2(b)).

### 3.3. Subgroup Analysis of TACE Combined with Sorafenib Group and TACE Group

**Figure 3:**
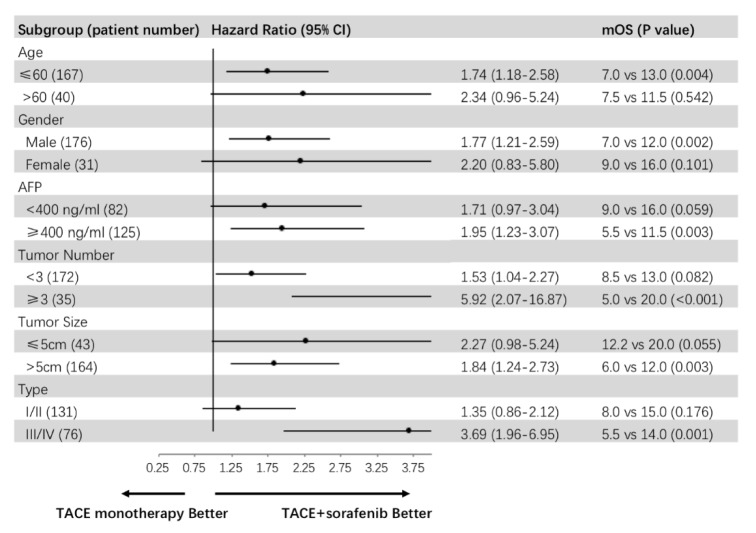
Subgroup analyses: The younger patients (≤60 years old), the male patients, and the patients with AFP more than 400ng/ml, tumor size more than 5cm, or type III/IV PVTT, the median OS of TACE + sorafenib group was longer than that of TACE alone.

To further define which subgroups patients could get benefits from the combined sorafenib treatment, subgroup analysis was conducted and found that, regardless of tumor number (<3 or ≥3), the efficiency of TACE combined with sorafenib treatment was always superior to the TACE alone. For the younger patients (≤60 years old), the male patients, and the patients with AFP more than 400ng/ml, tumor size more than 5cm, or type III/IV PVTT, the median OS of TACE combined with sorafenib group was longer than that of TACE alone. In addition, we investigated if there was a significant difference between the two groups with different Child-Pugh classification in median OS. We found that, in patients with Child-Pugh A, the efficiency of TACE combined with sorafenib treatment was superior to the TACE alone. However, the number of patients with Child-Pugh B was too small to further analyse (Figure 3).

## 4. Discussion

PVTT is known as the independent risk factor for the prognosis of HCC, and by now there is still an unmet obstacle in advanced stage HCC. According to the European and American guidelines, sorafenib therapy was recommended for HCC patients with BCLC C stage. Two large clinical trials including SHARP study and oriental study had verified the effectiveness of sorafenib which could improve the survival time for HCC patients about 3 months. Subgroup analysis showed that, whether combined with vascular invasion or not, sorafenib group had significantly better survival compared with placebo group. But the objective response rates (ORR) in the two trials were 2% and 3.3 %, suggesting that the efficacy of sorafenib remains limited [8, 9].

However, in Asia, TACE is still the main treatment method with clear curative effect. Several researches compared the prognoses between TACE and conservative treatment and indicated that TACE could improve the OS of HCC patients with PVTT. A prospective comparative study that included 164 patients showed that the TACE group had significantly better survivals than the conservative group, and the 1- and 2-year overall survival rates were 30.9%, 9.2%, and 3.8%, 0%, respectively (P<0.001). On subgroup analysis of segmental and major PVTT, the TACE group also had significantly better survivals (P = 0.002, P = 0.002) [10]. A meta-analysis that included 1601 patients revealed that TACE significantly improved the 6-month and 1-year overall survival of patients with PVTT compared with conservative treatment (P=0.001, P=0.001). Subgroup analyses showed that TACE was significantly effective whether with major or branch PVTT. The most frequent complication of TACE was postembolization syndrome, including fever, nausea, vomiting, and abdominal pain. Fatal complications including hepatic failure, spontaneous peritonitis, and gastrointestinal bleeding were rare.

Nevertheless, the disadvantage of TACE still remains, that TACE may lead to increased secretion of VEGF which perhaps rises the recurrence rate of HCC [11]. What is more, repeated TACE could increase the incidence of hepatic failure in the patients with PVTT. Sorafenib is a multikinase inhibitor which processes antiangiogenesis via inhibiting VEGFR, thus antagonizing the proangiogenesis VEGF after TACE when combined with sorafenib.

In our study, we found that the OS were significantly different between TACE combined with sorafenib group and TACE group. A propensity score matching was constructed to reduce the potential bias of baseline demographic and clinical characteristics between the two groups; TACE combined with sorafenib still had significantly better survival in HCC with PVTT. Subgroup analysis showed that, for the younger patients (≤60 years old), the male patients, or patients with AFP more than 400ng/ml, tumor size more than 5cm, or type III/IV PVTT, the median OS of combined therapy was longer than that of TACE, indicating that patients with these characteristics were more suitable for TACE combined with sorafenib therapy. The results indicated that patients with more tumor burden may get survival benefit from combination therapy. A retrospective controlled study that included 91 patients showed that TACE combined with sorafenib had significant better survival compared with TACE. Subgroup analysis revealed that, in patients with type I/II PVTT, combination therapy showed significant survival benefits (P=0.002, P=0.003), but in patients with type III PVTT, the OS between the two groups is similar (p=0.588) [12]. A multicenter, propensity matching score analysis showed similar result: TACE combined with sorafenib achieved more survival benefits in branch PVTT than main branch PVTT [13]. In our series, the more survival benefit was obtained in the patients with type III/IV PVTT which usually had poor response to TACE. The inconsistent results need to be further confirmed in the prospective rigid designed control study.

As a retrospective study, there are some limitations for the presence of possible bias; further prospective randomized trial (RCT) is encouraged to get high gradient evidence to evaluate the combination effect in HCC patients with PVTT.

## 5. Conclusions

Compared with TACE therapy alone, TACE combined with sorafenib could improve OS in HBV background HCC patients with PVTT. The patients who are younger, male, or with more tumor burden may benefit more from combination therapy.

## Figures and Tables

**Table 1:** Baseline demographic and clinical characteristics of the patients (n=498).

Characteristic	TACE+sorafenib (n=69)	TACE (n=429)	X^2^/t/z Value	P Value
Age	51 (21-79)	51 (18-84)	-0.431	0.667
Sex				
Male	59	380	0.537	0.464
Female	10	49		
Child-Pugh				
A	67	406	0.756	0.385
B	2	23		
AFP	12307.24±20255.88	19716.23±25103.12	-2.117	0.034*∗*
Number				
<3	58	368	0	0.985
≥3	11	68		
Size	8.39±4.45	9.65±3.25	-2.020	0.043*∗*
Type				
I/II	43	182	9.499	0.002*∗*
III/IV	26	247		
Treatment after TACE				
surgery	1	5	0.014	0.993
RFA	1	6		
radiotherapy	2	11		

*∗*p<0.05

**Table 2:** Baseline demographic and clinical characteristics of two propensity-matched groups (n=207).

Characteristic	TACE+sorafenib (n=69)	TACE (n=168)	X^2^/t/z Value	P Value
Age	50.85±11.52	51.4±11.20	-0.357	0.722
Sex				
Male	59	117	0.019	0.890
Female	10	21		
Child-Pugh				
A	67	133	0.074	0.786
B	2	5		
AFP	12307.24±20255.88	13774.00±21995.32	-0.464	0.343
Number				
<3	58	173	0.009	0.925
≥3	11	34		
Size	8.39±4.45	8.55±3.39	-0.285	0.795
Type				
I/II	43	88	0.042	0.838
III/IV	26	50		
Treatment after TACE				
surgery	1	2	0.085	0.958
RFA	1	3		
radiotherapy	2	6		

*∗*p<0.05

## Data Availability

The clinical data used to support the findings of this study are available from the corresponding author upon request.
